# The effect of psychotherapy on anxiety, depression, and quality of life in patients with diabetic retinopathy: A protocol for systematic review and network meta-analysis

**DOI:** 10.1097/MD.0000000000028386

**Published:** 2021-12-23

**Authors:** Suiping Li, Hong Liu, Xian Zhu

**Affiliations:** a Department of Endocrinology, Zhuzhou Central Hospital, Zhuzhou, Hunan Province, China; b Department of Nuclear Medicine, Zhuzhou Central Hospital, Zhuzhou, Hunan Province, China.

**Keywords:** Anxiety, Depression, Diabetic retinopathy, Network meta-analysis, Protocol, Psychotherapy, Quality of life

## Abstract

**Background:**

Diabetic retinopathy (DR) is one of the common microvascular complications in diabetic patients, which is characterized by progressive development and often leads to irreversible visual impairment. More seriously, DR patients suffer great psychological stress due to impaired visual function and reduced self-care ability such as anxiety and depression, which seriously affect the quality of life of patients. In recent years, various psychological therapies have been applied to alleviate anxiety, depression, and quality of life in DR patients, which have achieved positive results. However, the effectiveness and safety of different psychological therapies are unclear. This study aims to assess the effects of psychotherapy on anxiety, depression, and quality of life in DR patients through a network meta-analysis.

**Methods:**

All randomized controlled trials (RCTs) on the effects of psychotherapy on anxiety, depression, and quality of life in patients with DR published before 30 November 30, 2021, will be searched in the PubMed, EMBASE, Cochrane Register of Controlled Trials, Web of Science, Chinese Scientific Journal Database, China National Knowledge Infrastructure Database, Wanfang, and China Biomedical Literature Database. There will have no restrictions on publication status and language. Two researchers will be independently responsible for RCT screening, data extraction, and quality evaluation. All statistical analyses will be performed using Stata 14.0 and R 4.1.2.

**Results:**

The results of this meta-analysis will be submitted to a peer-reviewed journal for publication.

**Conclusion:**

This study will provide comprehensive and reliable evidence-based references for elucidating the efficacy of psychotherapy on anxiety, depression, and quality of life in DR patients.

**Ethics and dissemination:**

Ethical approval was not required for this study. The systematic review will be published in a peer-reviewed journal, presented at conferences, and shared on social media platforms.

**OSF Registration number:**

DOI 10.17605/OSF.IO/K8T27.

## 1 Introduction

Diabetic retinopathy (DR) is the most serious vascular disease of the eye caused by diabetes.^[1]^ Retinal damage stimulates the growth of new blood vessels and causes fibrous tissue proliferation. It leads to retinal detachment and vitreous hemorrhage, vision loss and even complete blindness in severe cases, which serious influences daily life, self-care, and psychological and emotional well-being.^[2,3]^ Current treatment of DM is complex, with a long course and unsatisfactory efficacy. DM-induced visual impairment brings great psychological stress that seriously affects the quality of life.^[4,5]^ It is reported that DR patients are prone to adverse emotional reactions such as anxiety and depression.^[6–8]^ It not only threatens the physical and mental health of DR patients, but also reduces the quality of life of patients.^[9–12]^ Therefore, how to alleviate anxiety and depression, improve the quality of life, and enhance the therapeutic efficacy on DR has been well concerned.

At present, the biological medicine model has gradually transformed into the biology-psychology-sociology medicine model. Somatic diseases with psychological problems have been well concerned not only by medical and psychological field, but also patients themselves.^[13]^ Treatment of DR alone, rather than a combination with psychological intervention, may aggravates it or causes psychological complications, which eventually results in a poor prognosis.^[14]^ Assessment of the psychological status of DR patients helps identify their psychological problems, and provides appropriate and targeted psychological interventions to improve the quality of life and clinical outcomes.^[15–17]^ In recent years, psychotherapy has been well appreciated, which has been applied to DR patients and achieved acceptable outcomes.^[18–22]^

Network meta-analysis (NMA) is the theory extension of traditional meta-analysis, which compares multiple treatments simultaneously in a single systematic evaluation, thus providing better clinical decisions about drug selection. Therefore, NMA is believed as a powerful tool to compare the effectiveness of multiple studies.^[23]^ Due to the wide variety and different therapeutic focuses of psychotherapies, how to compare the efficacy and safety of psychotherapies is challenging. In this study, we will perform NMA to analyze the effects of psychotherapy on anxiety, depression, and quality of life in patients with DR via analyzing relevant RCTs, thus providing references for clinical practice.

## 2 Methods

### 2.1 Study registration

This study has been registered in the OSF Registries (OSF registration number: DOI 10.17605/OSF.IO/K8T27), which follows the statement guidelines of preferred reporting items for systematic reviews and meta-analyses protocol.^[24]^

### 2.2 Eligibility criteria of inclusion of studies

#### 2.2.1 Types of studies

RCTs reporting the application of psychotherapy on anxiety, depression, and quality of life in DR patients.

#### 2.2.2 Types of participants

Patients diagnosed with DR.

#### 2.2.3 Types of interventions

One or more psychotherapy interventions, such as mindfulness therapy, cognitive-behavioral therapy, meditation therapy, comprehensive self-control training, acceptance and commitment therapy, and behavioral activation performed in experimental group. Conventional care measures or other types of interventions performed in control group.

#### 2.2.4 Types of outcome measures

1. Anxiety evaluated by the Self-Rating Anxiety Scale (SAS) and Hamilton Anxiety Scale (HAMA);
2. Depression evaluated by the Hamilton Depression Rating Scale (HAMD), Beck Depression Inventory (BDI), and Self-Rating Depression Scale (SDS);
3. Quality of life evaluated by the Medical Outcomes Study Short-Form 36 and World Health Organization Quality of Life Scale.

### 2.3 Exclusion criteria

1. Duplicate publications;
2. Studies with incomplete data;
3. Studies with inconsistent outcomes.

### 2.4 Data sources

All RCTs on the effects of psychotherapy on anxiety, depression, and quality of life in patients with DR published before November 30, 2021, will be searched in the PubMed, EMBASE, Cochrane Register of Controlled Trials, Web of Science, Chinese Scientific Journal Database, China National Knowledge Infrastructure Database, Wanfang, and China Biomedical Literature Database through searching MeSH terms and key words. There will have no restrictions on publication status and language. In addition, references of eligible literatures will be manually searched to avoid any missing.

### 2.5 Searching strategy

Search strategy in the PubMed is listed in Table 1. Literature search in other online databases will be similarly conducted.

**Table 1:** Search strategy used in PubMed.

Number	Search terms
#1	Diabetic Retinopathy[MeSH]
#2	Diabetic Retinopathies[Title/Abstract]
#3	Retinopathies, Diabetic[Title/Abstract]
#4	Retinopathy, Diabetic[Title/Abstract]
#5	OR/1–4
#6	Psychotherapy[MeSH]
#7	Logotherapy[Title/Abstract]
#8	Logotherapies[Title/Abstract]
#9	Psychotherapies[Title/Abstract]
#10	Cognitive Therapy[MeSH]
#11	Behavior Therapy, Cognitive[Title/Abstract]
#12	Psychotherapy, Cognitive[Title/Abstract]
#13	Cognition Therapy[Title/Abstract]
#14	Cognitive Behavior Therapy[Title/Abstract]
#15	Cognitive Behavioral Therapy[Title/Abstract]
#16	Cognitive Psychotherapy[Title/Abstract]
#17	Therapy, Cognition[Title/Abstract]
#18	Therapy, Cognitive[Title/Abstract]
#19	Therapy, Cognitive Behavior[Title/Abstract]
#20	Behavior Therapies, Cognitive[Title/Abstract]
#21	Behavioral Therapies, Cognitive[Title/Abstract]
#22	Behavioral Therapy, Cognitive[Title/Abstract]
#23	Cognition Therapies[Title/Abstract]
#24	Cognitive Behavior Therapies[Title/Abstract]
#25	Cognitive Behavioral Therapies[Title/Abstract]
#26	Cognitive Psychotherapies[Title/Abstract]
#27	Cognitive Therapies[Title/Abstract]
#28	Psychotherapies, Cognitive[Title/Abstract]
#29	Therapies, Cognition[Title/Abstract]
#30	Therapies, Cognitive[Title/Abstract]
#31	Therapies, Cognitive Behavior[Title/Abstract]
#32	Therapies, Cognitive Behavioral[Title/Abstract]
#33	Therapy, Cognitive Behavioral[Title/Abstract]
#34	Mindfulness[Title/Abstract]
#35	Mindfulness therapy[Title/Abstract]
#36	Meditation therapy[Title/Abstract]
#37	Comprehensive self-control training[Title/Abstract]
#38	Cceptance and commitment therapy[Title/Abstract]
#39	Behavioral activation [Title/Abstract]
#40	OR/6-39
#41	Randomized Controlled Trials as Topic[MeSH]
#42	Clinical Trials, Randomized[Title/Abstract]
#43	Controlled Clinical Trials, Randomized[Title/Abstract]
#44	Trials, Randomized Clinical[Title/Abstract]
#45	Random∗[Title/Abstract]
#46	OR/41-45
#47	#5 AND #40 AND #46

### 2.6 Data collection and analysis

#### 2.6.1 Literature screening and data extraction

Retrieved literatures will be imported into EndNote for automatic weighting combined with manual weighting to ensure the exclusion of duplicate literatures. Literature screening will be independently performed by 2 researchers based on inclusion and exclusion criteria, with final cross-checking of inclusion results. A third researcher will be involved in case of disagreement.

**Figure 1:**
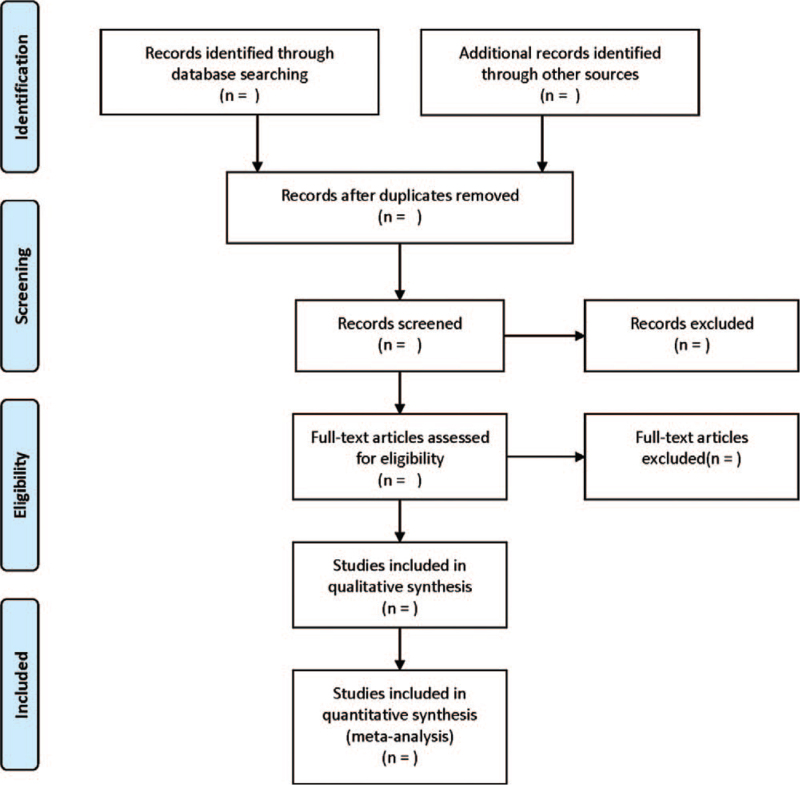
Flow diagram of studies identified.

The following data will be extracted from each eligible literature: Baseline characteristics of literatures: title, first author, year of publication, and source; Baseline characteristics of subjects: gender, age, race, and case number; Interventions: types of psychotherapy and intervention details in experimental group, and intervention details in control group; Risk assessment of RCT bias: 7 aspects of the literature, including random sequence generation, allocation concealment, implementation of blinding, whether blinding is applied to outcome assessment, completeness of outcome data, whether results are selectively reported, and whether other biases are present; and Outcome indicators: types of scales, scores before and after intervention. The study flow diagram is shown in Figure 1.

#### 2.6.2 Assessment of evidence quality

Evidence quality of included studies will be evaluated according to the Risk of Bias Assessment Tool recommended by Cochrane5.1, including the random sequence generation, allocation concealment, implementation of blinding, whether blinding is applied to outcome assessment, completeness of outcome data, whether results are selectively reported, and whether other biases are present.^[25]^ RevMan 5.3 software will be used to plot the quality of the literature evaluation.

#### 2.6.3 Measures of therapeutic efficacy

For continuous variables, standardized mean differences (SMD) and corresponding 95% confidence intervals (CIs) will be used to calculate the effect size.

#### 2.6.4 Management of missing data

Missing data will be requested by emailing the first author. If the missing data cannot be obtained, they will be excluded from the study.

### 2.7 Data analyses

#### 2.7.1 Network Meta-analysis

NMA will be performed using Stata 14.0 and R 4.1.2. R.4.1.2 software was used to call the “gemtc” and “rjags” packages for statistical analysis, with 4 chains with a refinement iteration step size of 10 and 50,000 iterations, and the first 10,000 iterations of the annealing algorithm will be used to eliminate the effect of the initial values. Convergence will be evaluated to assess the robustness of the results. The network model constructed for each outcome indicator will be tested for its heterogeneity, and the magnitude of heterogeneity will be quantitatively determined by combining the mesh model *I*^2^ values, in which, *I*^2^ > 50% suggested a large heterogeneity among included literatures, and the source of heterogeneity should be analyzed. The network relationships will be mapped using Stata 14.0. Inconsistency tests will be performed in case of a closed loop between interventions, and inconsistency factors (IFs) will be calculated, and judgments will be made based on the *P* values of IF and Z test. *P* > .05 and the starting point of 95% CI of IF at 0 suggests the consistency between direct and indirect comparison. Pairwise comparison tables will be presented, surface under the cumulative ranking curves (SUCRA) values will be calculated, and cumulative probability ranking will be plotted.

#### 2.7.2 Assessment of publication biases

Comparison-adjusted funnel plots will be plotted to assess the publication bias and small-sample effects.^[26]^

#### 2.7.3 Subgroup analysis

Subgroup analyses will be performed based on the intervention time, the type of scale, and the age of the patient.

#### 2.7.4 Sensitivity analysis

The stability of the results of the meta-analysis will be tested by sensitivity analysis using the one-by-one elimination method.

#### 2.7.5 Ethics and dissemination

The content of this article did not involve moral approval or ethical review and would be presented in print or at relevant conferences.

## 3 Discussion

DR is the most common cause of newly onset blindness in adults, which causes anxiety and depression, and severely affects the quality of life.^[27–29]^ Effective and appropriate psychological interventions for DR patients can reduce their psychological stress, promote psychological recovery, and improve quality of life and treatment outcomes.^[30]^ Psychotherapy has received a widespread attention because of its low incidences of adverse effects. Although psychotherapy can improve the psychological problems such as anxiety and depression, and improve the quality of life of DR patients, we unclear which one is the optimal because of the numerous psychotherapies and different therapeutic focuses. Therefore, it is difficult to determine which psychotherapy presents the best efficacy and safety. This study aims to investigate the effects of different psychological therapies on alleviating anxiety, depression, and quality of life in patients with DR through a NMA, thus providing an evidence-based basis for making the best clinical decisions.

Our NMA has some limitations. First, due to publication language limitations, we will only include literatures published in English and Chinese, which is likely to lead to a risk of bias. Second, different intervention methods, intervention duration, and measurements of various psychotherapies, as well as limitations in the quality of literatures may increase the possibility of heterogeneity. In fact, our findings will provide evidence to support the best psychotherapy on alleviating anxiety, depression, and quality of life in DR patients.

## Author contributions

**Data curation:** Suiping Li.

**Formal analysis:** Xian Zhu.

**Methodology:** Hong Liu.

**Project administration:** Xian Zhu.

**Software:** Suiping Li.

**Supervision:** Hong Liu.

**Validation:** Xian Zhu.

**Visualization and software:** Suiping Li.

**Visualization:** Suiping Li.

**Writing – original draft:** Suiping Li and Xian Zhu.

**Writing – review & editing:** Suiping Li and Xian Zhu.
